# The effect of social support on college students’ sports anomie behavior: the mediating role of self-efficacy and attitude’s regulating role in sports normative behavior

**DOI:** 10.3389/fpsyg.2025.1662985

**Published:** 2025-09-25

**Authors:** Liping Liu, Yuqing Yang, Han Liu, Shanping Chen, Yao Shang, Yifei Song

**Affiliations:** ^1^School of Physical Education, Xi’an Jiaotong University, Xi’an, China; ^2^Xingzhi College of Xi’an University of Finance and Economics, Xi’an, China

**Keywords:** social support, sports anomie behavior, self-efficacy, regulate attitude, college students

## Abstract

**Background:**

College students’ sports anomie behavior negatively impacts their physical health, undermines the implementation of the educational philosophies of Fostering Virtue through Education and Health First. There is a lack of quantitative research on the mechanisms underlying such behavior. Based on the Conservation of Resources Theory, this study aims to examine the mechanism through which social support influences college students’ sports anomie behavior.

**Methods:**

A total of 2,340 students from 20 universities across China were selected using stratified sampling. Data were collected using the Social Support Scale and the Sports Anomie Behavior Brief Inventory. Mediation and moderation effects were analyzed using AMOS 28.0, SPSS 24.0, and the Process plugin.

**Results:**

Social support significantly negatively predicted physical misconduct. Physical norm self-efficacy fully mediated the relationship between social support and physical misconduct. Attitudes toward physical norm behavior significantly moderated both the antecedent and subsequent paths of the mediation model.

**Conclusion:**

The study validates a moderated mediation model of “social support → physical norm self-efficacy→sports anomie behavior.” Universities should establish a multi-support network involving parents, teachers, and peers, and integrate normative education into physical education curricula. These efforts should synergistically enhance students’ attitudes toward physical norms and self-efficacy from external to internal levels, thereby reducing sports anomie behavior and promoting physical health.

## 1 Introduction

College students’ sports anomie behavior refers to a series of behavioral manifestations in which students violate social norms—such as sports ethics, sports competition rules, sports regulations, and laws and regulations—in the process of participating in sports activities (Chen et al., 2021). The sports anomie behavior of college students has attracted scholars’ attention. Sports anomie behavior negatively impacts the physical health of about 38.33 million college students in China. Moreover, it is not conducive to the educational concept of “implementing the fundamental task of cultivating morality and the guiding ideology of health first, and promoting the health and all-round development of students.” In recent years, sports anomie behavior research related to college students has progressed, from initial simple phenomenon description and reason to contemporary quantitative research, especially in theoretical models, scale development, and status quo characteristics, which have provided useful results. However, the lack of research on sports anomie behavior and its influence mechanism on college students is not conducive to targeting intervention suggestions to prevent and reduce their sports anomie behavior.

Global scholars’ research on factors influencing college students’ sports behavior mainly concerns personal and social factors. Social support, self-efficacy, and attitude are important factors affecting college students (Meng and Zhang, 2022). Previous studies have been mainly limited to the influence on positive sports behavior. Still, as a negative expression of sports behavior, sports anomie behavior has not explored the influence mechanism among the three. Social support and sports behavior are inseparable. Suppose family members, friends, and classmates can provide material and spiritual security support for college students to exercise. In that case, the confidence and emotional experience of college students in physical exercise will be enhanced, which is conducive to promoting the formation of healthy behaviors and habits. It is not easy to have sports anomie behavior.

At the same time, according to research on college students’ self-efficacy, when college students feel support and care from their families, teachers, and friends, they will believe that they are more likely to achieve their exercise goals and tend to be more active, committed to, and passionate in sports activities (Dong et al., 2018). They will demonstrate more standard sports behavior, indicating that self-efficacy may play an important role in the relationship between social support and sports anomie behavior. Previous studies have constructed psychological mechanism models of sports anomie behavior in sports based on psychological factors such as behavioral motivations, behavioral attitudes, and self-efficacy. However, qualitative analysis of these constructs and theoretical assumptions remains insufficient, necessitating further quantitative research on college students’ deviant behavior in sports and its psychological mechanisms. Attitudes toward sports normative behavior refer to college students’ evaluative judgments regarding the consequences of complying with or violating sports behavioral norms. This encompasses assessments of both anomie and norm-compliant behaviors, and the evaluative orientation should also include both favorable and unfavorable aspects (Chen et al., 2023a). In addition, it is difficult for college students to take the first step of observing sports standard behavior because of their varying attitudes towards sports standard behavior.

Sports behavior attitude is also an important variable affecting the sports behavior of college students. Based on the conservation of resources theory, the study discusses the influence on sports anomie behavior of social support on anomie behavior in sports among ordinary college students. In this study, the self-efficacy of college students’ compliance with sports norms as the intermediary variable and the attitude of sports norms behavior as the regulating variable to reveal the action mechanism to more profoundly describe the process of social support on sports anomie behavior and provide empirical basis for preventing and reducing such behavior in ordinary universities is the focus.

## 2 Theoretical basis and research hypotheses

### 2.1 Theoretical basis

The conservation of resources theory was first proposed in 1989 by Hobfoll (1989). It describes how employees work tirelessly to obtain, maintain, nurture, and protect what they consider valuable resources, including individual resources and relational resources (Hobfoll, 2001). Individual resources refer to the individual’s optimistic personality, healthy psychology, and strong self-efficacy. Relationship resources are independent of individuals and exist in social interactions, such as social and organizational support, partners’ health, and so on (Hobfoll, 2002). The core principle of the conservation of resources theory includes the loss first principle. Individuals will take measures to avoid the loss of resources. The resource investment principle reveals how individuals can invest in existing resources to obtain new resources to cope with possible future stress situations. Finally, the paradox principle indicates that for individuals, the fewer resources they have, the more important it is for the infusion and increase of resources to relieve tension and stress (Halbesleben et al., 2014). The applicability and validity of the theory have been fully tested and confirmed in the field of organizational behavior and education (Zhou and Ning, 2020). This theory has important reference value for explaining many psychological behavior processes in college students’ physical exercise. Students’ sports behavior is inseparable from the degree of social support. As an important relationship resource for students, social support affects students’ individual resources, especially student’ self-efficacy.

Social support provides students with various types of support and training needed for physical exercise, so that they have an objective cognition of the difficulty of physical exercise. It also reduces students’ worries in the face of physical exercise. Students feel that their exercise resources are better, and will be more confident in physical exercise and more likely to maintain positive physical exercise behavior.

At the same time, students with poor relationship resources also have lower perceived levels of social support. They are more inclined to reduce exercise and emotional input to prevent resource loss. They no longer have confidence in physical exercise because they perceive the threat of losing related resources and feel their efforts cannot achieve the expected exercise effect. Consequently, negative physical behavior may occur. We believe the direction of social support on students’ sports anomie behavior may be “social support-self-efficacy-physical education behavior.”

According to the conservation of resources theory, sports normative attitude, as students’ individual resources, may influence the relationship between social support and students’ sports behavior. Specifically, students need to invest to prevent the future loss of resources. However, resource status can affect student choice: fully resourced students are more inclined to make active investments due to their ability and confidence to acquire other individual resources and relationships. Students with fewer resources may choose to retain existing resources due to excessive concern about resource loss.

When students have a positive attitude towards sports norms, their resources are relatively rich. Hence, they are more willing to put these resources into physical exercise, show more active exercise behavior, and solve problems encountered in the exercise process. At the same time, it is not easy to be restricted by exercise resources. Conversely, when students have a negative attitude towards sports norms, this uncertainty triggers defensive psychology, leading to a tendency to retain resources rather than invest in exercise. This means resources provided by social support may not be used by students in physical exercise. Physical normative attitudes moderate the influence of social support on physical behavior.

### 2.2 Study hypotheses

Social support can significantly improve individuals’ self-restraint ability. When individuals feel their families, friends, or teachers care and support, they are more likely to resist temptation and better follow sports norms (Dong, 2017). In the social support environment, the supervision mechanism can be formed to restrain and regulate individual behavior and find and correct anomic behavior in time (Liu et al., 2020). Additionally, the role model of the people around them and the healthy sports culture atmosphere make it easier for individuals to feel the social value and significance of abiding by sports norms, thus gradually forming good sports ethics habits and reducing the occurrence of anomie behavior. Social support also provides individuals with the necessary resources and support to follow norms in physical activity better (Zhang and Wang, 2021). Based on the above studies, we believe sports anomie behavior is closely related to the degree of social support. Social support can provide important spiritual and material resources for college students’ physical exercise behavior, which is conducive to forming positive physical exercise behavior and habits and reducing the occurrence of sports anomie behavior. Accordingly, the study proposes Hypothesis 1 (H1): Social support negatively predicts the sports anomie behavior of college students.

As an important psychological decision variable in physical behavior, self-efficacy is an individual’s expectation of successfully adhering to physical exercise in various situations. As an important relationship resource for students, social support conversely affects students’ individual resources, especially students’ self-efficacy. Social support provides students with various types of support and help needed for physical exercise, so that students have objective cognition of physical exercise difficulties and reduce worry related to physical exercise. When students feel that their exercise resources are better, they will be more confident in physical exercise and are more likely to maintain positive physical exercise behavior. At the same time, according to the cognitive decision theory of sports behavior, the higher the confidence in the completion behavior, the stronger the willingness to adopt the behavior, the more likely the behavior is to occur (Chen and Li, 2007; Chen et al., 2014). Specifically, to the psychological mechanism of college students’ sports anomie behavior, the higher the self-efficacy of students to abide by the behavior norms in sports activities, the more likely they are to abide by the behavior norms, reducing sports anomie behavior.

In conclusion, this study proposes Hypothesis 2a (H2a): Social support directly affects the self-efficacy of sports norms. Hypothesis 2b (H2b): Sports regulation self-efficacy has a direct negative effect on sports norms. According to the relationship revealed by hypotheses H2a and H2b, we further propose Hypothesis 2 (H2): Sports regulation self-efficacy mediates between social support and sports anomie behavior.

According to the conservation of resources theory, the attitude of sports normative behavior as college students’ individual resources may influence the relationship between social support and college students. College students must invest to prevent the possible loss of future resources. However, resource status will affect college students’ investment. College students with more resources tend to choose more active investments because they have the ability and confidence to obtain other individual and relationship resources.

In contrast, students with fewer resources will usually choose to save their existing resources because of exaggerating the possibility of resource loss. When college students have a more positive attitude towards sports and standard behavior, and are more capable and confident in obtaining other individual resources and relationship resources (social support), they will invest more actively in resources and be more willing to use their individual resources for physical exercise. Accordingly, we propose Hypothesis 3a (H3a): Sports normative behavior attitudes play a positive regulatory role in the relationship between social support and self-efficacy.

On the contrary, when students have a low attitude towards physical education behavior, the importance and incomprehension of physical activity behavior can easily trigger defensive psychology. Hence, they tend to save resources in exercise rather than invest in resources. This means that the resources of social support may not be used by students in physical exercise, and the attitude of physical normative behavior influences the positive effect of social support on physical behavior, (Liu and Xu, 2023) which easily leads to sports anomie behavior. Therefore, we propose Hypothesis 3b (H3b): Sports normative behavior attitude positively regulates the relationship between social support and sports anomie behavior.

**Figure 1:**
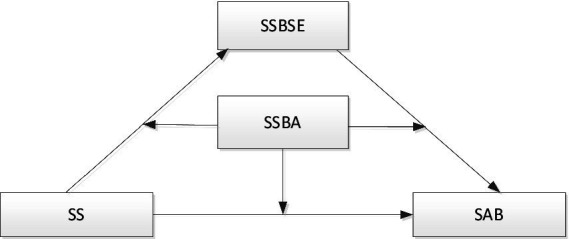
Analytic framework. SS represents “social support,” SSBA represents “sports standard behavior attitude,” SAB represents “sports anomie behavior,” and SSBSE represents “sport specification behavior self-efficacy.” The same holds true for the below.

According to the cognitive decision theory of sports behavior, individuals believe that a satisfactory effect will strengthen behavioral intention, further leading to the repetition of this behavior (Chen et al., 2014). We apply this view to the psychological mechanism of college students’ sports anomie behavior. Suppose students think that obeying the code of behavior in sports activities will bring benefits, and violating the code of behavior will bring disadvantages. In that case, they will tend to obey the code of behavior, and sports anomie behavior will be reduced. On the contrary, if students think there is no benefit from observing sports activities’ code of behavior, and violating the code of behavior will bring benefits, they will have the intention of violating the code of behavior, and physical anomie behavior will increase. Accordingly, the study proposes Hypothesis 3c (H3c): Sports normative behavior attitude plays a positive regulatory role in the relationship between self-efficacy and sports anomie behavior. The hypothetical framework for the above variables is shown in Figure 1.

## 3 Study subjects and methods

### 3.1 Study subjects

This study employed a stratified sampling approach involving 20 universities across China. The institutional distribution encompassed five universities in North China, two in Northeast China, five in East China, three in South Central China, three in Southwest China, and two in Northwest China. Within each participating institution, 120 questionnaires were systematically administered according to predetermined strata: 30 questionnaires per academic year level, with each year-level cohort comprising 15 male and 15 female respondents. Finally, a total of 2,400 undergraduate students completed the survey instruments, constituting the final research sample. 2,340 valid questionnaires were obtained, with an effective rate of 97.5%. Boys number 1145 (48.9%) and 1,195 are girls (51.1%). Freshmen number 598 (25.6%), 603 are sophomores (25.8%), 572 are juniors (24.4%), and 567 are seniors (24.2%).

### 3.2 Research tools

#### 3.2.1 Social support scale

The social support scale compiled by Chen Shanping is used in this part (Chen et al., 2021; Shang et al., 2025). The scale includes three statements: ① In sports, parents or other relatives can help me; ② In sports, teachers can help me; ③ My friends or classmates can help me. The Likert level 5 scale is used, from “strongly agree” to “strongly disagree,” from “1” to “5,” with all questions scored in reverse. The average score of the three questions is the score of the scale. The higher the score, the higher their social support level. In the Exploratory Factor Analysis (EFA), the KMO was 0.649 (>0.6), and Bartlett’s test of sphericity yielded the level of *p* = 0.000 (<0.05). Reliability analysis showed that the scale’s Cronbach’s *α* coefficient was 0.771, indicating good reliability.

#### 3.2.2 Sports anomie behavior scale

The sports anomie behavior scale of college students compiled by Chen Shanping is used in this part (Chen et al., 2023b). The scale includes five topics: ① Violation of the PE code of conduct, ② Violation of code of conduct in the Standard test, ③ Violation of behavior in extracurricular sports competitions, ④ Violation of the code of behavior in sports club activities, and ⑤ Violation of the code of conduct in extracurricular independent exercise. The Likert 5 scale is used to score from “never,” “rarely,” “sometimes,” “more,” to “often,” from “1” to “5.” The higher the score, the more anomie behavior incidents. In this part of the study, the Cronbach’s *α* coefficient of this scale is 0.947. The confirmatory factor analysis yielded the following results: χ^2^/df = 11.930, NFI = 0.988, CFI = 0.989, IFI = 0.989, TLI = 0.978, RMSEA = 0.068, indicating good structural validity of the scale.

#### 3.2.3 Self-efficacy scale of sports norms

The self-efficacy scale of sports norms compiled by Chen Shanping is used in this part (Chen et al., 2023a) and includes 15 questions with four dimensions: regularity, consciousness, interest, and convenience. Here we use a statement as an example: “Under any circumstances, I can stick to sportsmanship.” The Likert level 5 scale is used to score, from “agree” to “disagree,” from “1” to “5,” all questions are scored in reverse. The average score of the 5 questions is the score of the scale. The higher the score, the stronger the self-efficacy. In this part of the study, Cronbach’s *α* coefficient of this scale is 0.939. The confirmatory factor analysis yielded the following results: χ^2^/df = 16.033, NFI = 0.966, CFI = 0.968, IFI = 0.968, TLI = 0.948, RMSEA = 0.080, indicating good structural validity of the scale.

#### 3.2.4 The attitude towards sports norms Behaviors

The attitude towards sports norms behaviors scale compiled by Chen Shanping is used in this part (Chen et al., 2021). The scale includes 20 questions, including four dimensions: favorable compliance with norms, unfavorable compliance with norms, favorable anomie behavior, and unfavorable anomie behavior. A statement is used as an example: “Following the code of physical conduct is conducive to improving physical fitness.” The Likert level 5 scale is used to score, from “strongly agree” to “strongly disagree,” from “1” to “5.” Questions 1–5 and questions 16–20 are scored in reverse. A higher score indicates a more positive attitude towards sports norms. In this part of the study, the Cronbach’s *α* coefficient of this scale is 0.871, χ^2^/df = 10.520, NFI = 0.954, CFI = 0.958, IFI = 0.958, TLI = 0.945, RMSEA = 0.064, indicating good structural validity of the scale.

### 3.3 Study methods

Data are analyzed and processed using AMOS 28.0, SPSS 24.0, and its PROCESS macro plugin. The process macro program, written by Hayes in 2013, easily and effectively analyzes and processes a variety of structural equation models. It uses BC-Bootstrap interval estimation by default, a relatively robust operation method. The path model discussed in this study is a mixed model, which should be detectable by Process plug-in No.59. This model needs to test the regulatory effect of the front and posterior paths of the mediation effect, and the above two regulatory effects must exist before the model can be established. To detect regression significance, the study calculates repeated sampling 5,000 times using the 95% interval estimation of BC-Bootstrap, and the confidence interval contains no zero, indicating a significant effect.

## 4 Study results

### 4.1 The common method variance and test of distinguishing validity of variables

Common method variance (CMV) analysis is conducted using the Harman univariate test method. The cumulative variance interpretation rate after the first factor rotation is 26.897%, which is lower than the critical standard (40%) (Podsakoff et al., 2003). Secondly, AMOS 28.0 is used to build a structural equation model. We use the comparative fitness index (CFI), standardized root mean square residual (SRMR), and Tucker-Lewis index (TLI) to test the fitness of the model. A good fit is indicated when CFI is greater than 0.9. The closer CFI is to 1, the better the model fit. SRMR less than 0.05 represents a good model fit. According to the above criteria, all the items are added as a latent variable to the structural equation model. The model fit is poor: χ^2^/df = 104.228, CFI = 0.539, TLI = 0.468, SRMR = 0.1996, and did not meet acceptability criteria. Consequently, this study has no serious problem of common methodological bias.

Confirmatory factor analysis is used to test the benchmark model and other competitive alternative models. The results are shown in Table 1. The fits of the assumed four-factor model show the best performance, indicating a very good discriminatory validity between the four variables.

**Table 1:** The comparison of measurement model.

Model	*χ^2^/df*	SRMR	CFI	TLI
Benchmark Model: SS;SSBA;SAB;SSBSE	17.337	0.0460	0.931	0.916
Model 1: SS + SSBA+SAB + SSBSE	104.228	0.1996	0.539	0.468
Model 2: SS + SSBA+SAB;SSBSE	52.594	0.1492	0.772	0.734
Model 3: SS + SSBA+SSBSE;SAB	41.959	0.0956	0.819	0.789
Model 4: SS + SAB + SSBSE;SSBA	99.600	0.1972	0.563	0.491
Model 5: SS + SSBA;SAB;SSBSE	22.571	0.0852	0.906	0.889
Model 6: SS + SAB;SSBA;SSBSE	45.329	0.1272	0.808	0.771
Model 7: SS + SSBSE;SSBA;SAB	40.935	0.0940	0.827	0.794
Model 8: SSBA+SAB;SS;SSBSE	26.729	0.1091	0.888	0.867
Model 9: SSBA+SSBSE;SS;SAB	20.351	0.0640	0.916	0.900
Model 10: SS;SSBA+SAB + SSBSE	57.619	0.1550	0.366	0.302
Model 11: SAB + SSBSE;SS;SSBA	47.467	0.1400	0.481	0.427

SS represents “social support,” SSBA represents “sports standard behavior attitude,” SAB represents “sports anomie behavior,” and SSBSE represents “sport specification behavior self-efficacy.” The same holds true for the below.

### 4.2 Descriptive statistics and correlation analysis

Table 2 lists each variable’s mean, standard deviation, and correlation analysis results. The four core variables have a significant correlation and can be tested for subsequent tests.

**Table 2:** Description statistics and correlation analysis of each variable.

	M	SD	SAB	SS	SSBA	SSBSE
SAB	1.06	0.230	1			
SS	3.93	0.759	−0.066^**^	1		
SSBA	3.86	0.550	−0.205^***^	0.346^***^	1	
SSBSE	4.61	0.504	−0.234^***^	0.275^***^	0.420^***^	1

**p < 0.01; ***p < 0.001.

### 4.3 Mediation effect test

For the mediation effect test, social support is the independent variable, sports regulation self-efficacy is the mediation variable, sports anomie behavior is the dependent variable, and gender, grade, urban and rural areas, only child, key colleges, and class or league cadres are the control variables. Based on these variables, we test the mediating role of sports norm self-efficacy between social support and sports anomie behavior. Model 4 in Process is used to set 95% as confidence intervals, and 5,000 Bootstrap samples are repeated for mediation effect testing. The results are shown in Table 3, and the direct effect of social support on physical anomie behavior is significant (*β* = −0.012, *t* = −2.246, *p* < 0.05), with a Bootstrap 95% CI of [−0.021, −0.002], indicating Hypothesis 1 (H1) is correct. The influence of social support on the self-efficacy of sports norms is significant (*β* = 0.274, *t* = 13.665, *p* < 0.001), with a Bootstrap 95% CI of [0.235, 0.314]. The influence of physical normative self-efficacy on physical anomie behavior is significant (*β* = −0.053, *t* = −11.041, *p* < 0.001), with a Bootstrap 95% CI of [−0.062, −0.044]. The above points indicate Hypothesis 2a (H2a) and Hypothesis 2b (H2b) are valid. In the influence path “social support sports standard self-efficacy sports anomie behavior,” the direct effect of social support on sports anomie behavior is not significant (*β* = 0.003, *t* = 0.577, *p* > 0.05), with a Bootstrap 95% CI of [−0.007, 0.012].

**Table 3:** Test of the mediating role of sports norm self-efficacy between social support and sports anomie behavior.

	SAB			SSBSE			SAB		
	*β*	*t*	95%Cl	*β*	*t*	95%Cl	*β*	*t*	95%Cl
Controlled variable									
Gender	0.024	2.530^*^	[0.005,0.043]	−0.104	−2.583^**^	[−0.183,−0.025]	0.019	2.001^*^	[0.000,0.037]
Grade	0.018	4.238^***^	[0.010,0.026]	0.004	0.212	[−0.031,0.039]	0.018	4.395^***^	[0.010,0.026]
Urban	−0.017	−1.625	[−0.038,0.004]	0.055	1.217	[−0.034,0.143]	−0.015	−1.388	[−0.035,0.006]
Only Child	−0.009	−0.859	[−0.030,0.012]	−0.028	−0.615	[−0.116,0.061]	−0.011	−1.022	[−0.031,0.010]
Key Colleges	−0.016	−1.609	[−0.035,0.003]	0.029	0.705	[−0.051,0.109]	−0.014	−1.489	[−0.033,0.004]
Class or league cadres	−0.010	−1.078	[−0.029,0.009]	0.054	1.324	[−0.026,0.134]	−0.008	−0.803	[−0.026,0.011]
Independent variable									
SS	−0.012	−2.462^*^	[−0.021,−0.002]	0.274	13.665^***^	[0.235,0.314]	0.003	0.577	[−0.007,0.012]
SSBSE							−0.053	−11.041^***^	[−0.062,−0.044]
*R* ^2^	0.019			0.080			0.068		
*F*	6.532^***^			29.128^***^			21.248^***^		

*P < 0.05; **P < 0.01; ***P < 0.001.

Social support can directly influence sports anomie behavior, but after adding the intermediary variable of sports regulation self-efficacy, social support can completely influence sports anomie behavior through sports norm self-efficacy, indicating that Hypothesis 2 (H2) is verified.

### 4.4 The mediation effect test with regulation

**Figure 2:**
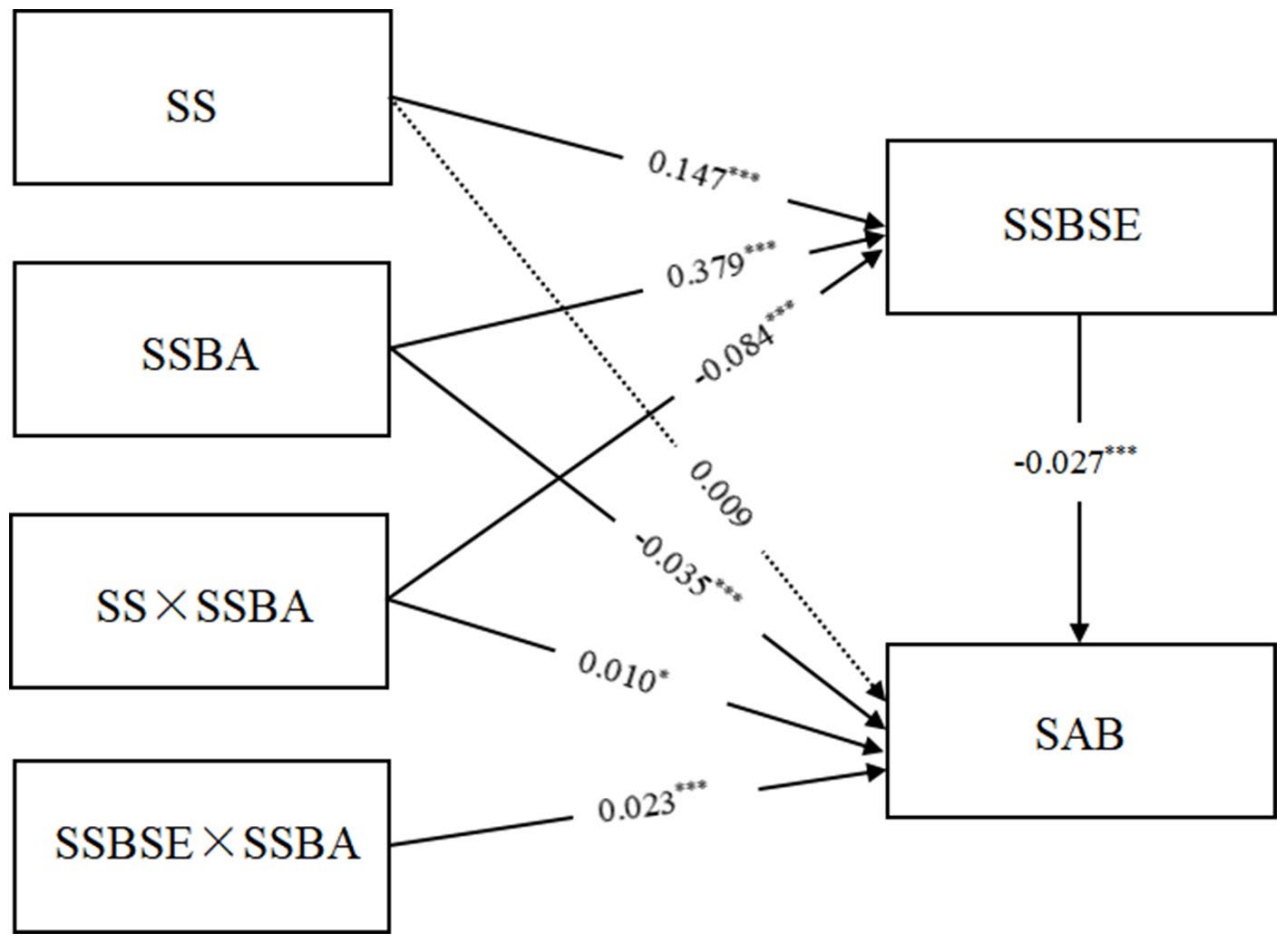
A mediation model of social support affecting sports anomie behavior with regulation.

The study takes social support as the independent variable, sports specification self-efficacy as the intermediary variable, attitude to sports norm behavior as the regulated variable, sports anomie behavior as the dependent variable, and takes gender, grade, urban and rural areas, only child, and key colleges and class or league cadres as the control variables, aiming to test the moderated mediating effect of social support promoting relationships. This is tested using the Process model 58. The results are shown in Table 4 and Figure 2. The attitude towards sports norm behavior positively predicts sports regulation self-efficacy [*β* = 0.379, *p* < 0.001, Bootstrap 95% CI: (0.340, 0.428)].

**Table 4:** Mediation model of sports normative behaviors attitude regulating sports anomie behavior.

Predictive variable	Model 1 (SSBSE)			Model 2 (SAB)		
	*β*	*t*	95%Cl	*β*	*t*	95%Cl
Gender	−0.045	−1.207	[−0.119,0.028]	0.015	1.613	[−0.003,0.033]
Grade	0.023	1.349	[−0.010,0.056]	0.017	4.194^***^	[0.009,0.025]
Urban	0.050	1.190	[−0.032,0.132]	−0.016	−1.589	[−0.037,0.004]
Only child	−0.032	−0.771	[−0.115,0.050]	−0.011	−1.060	[−0.031,0.009]
Key college	−0.000	−0.008	[−0.075,0.074]	−0.013	−1.403	[−0.031,0.005]
Class or league Cadres	0.019	0.492	[−0.056,0.093]	−0.006	−0.672	[−0.025,0.012]
SS	0.147	7.432***	[0.108,0.186]	0.009	1.861	[0.000,0.019]
SSBSE				−0.028	−4.667***	[−0.039,−0.016]
SSBA	0.379	18.993***	[0.340,0.418]	−0.035	−6.603***	[−0.046,−0.025]
SS*SSBA	−0.084	−4.765***	[−0.119,−0.050]	0.010	2.238*	[0.001,0.019]
SSBSE*SSBA				0.023	4.417***	[0.013,0.033]
*R* ^2^	0.206			0.093		
*F*	67.238***			21.652***		

*P < 0.05; **P < 0.01; ***P < 0.001.

There is significant interaction between social support and sports standard behavior attitude [*β =* −0.084, *p* < 0.001, Bootstrap 95% CI: (−0.119,-0.050)], indicating that sports norms behavior attitude regulates the influence of social support on the self-efficacy of sports norms - the first half of the intermediary. Sport specification behavior attitude significantly negatively predicts sports anomie behavior [*β* = −0.035, *p* < 0.001, Bootstrap 95% CI: (−0.046,-0.025)]. Sport specification self-efficacy and sports specification behavior attitude have a significant interaction effect [*β* = 0.023, *p* < 0.001, Bootstrap 95% CI: (0.013, 0.033)], indicating that sports norm behavior attitude regulates influence of sports norm self-efficacy on sports anomie behavior - the second half of the intermediary.

**Figure 3:**
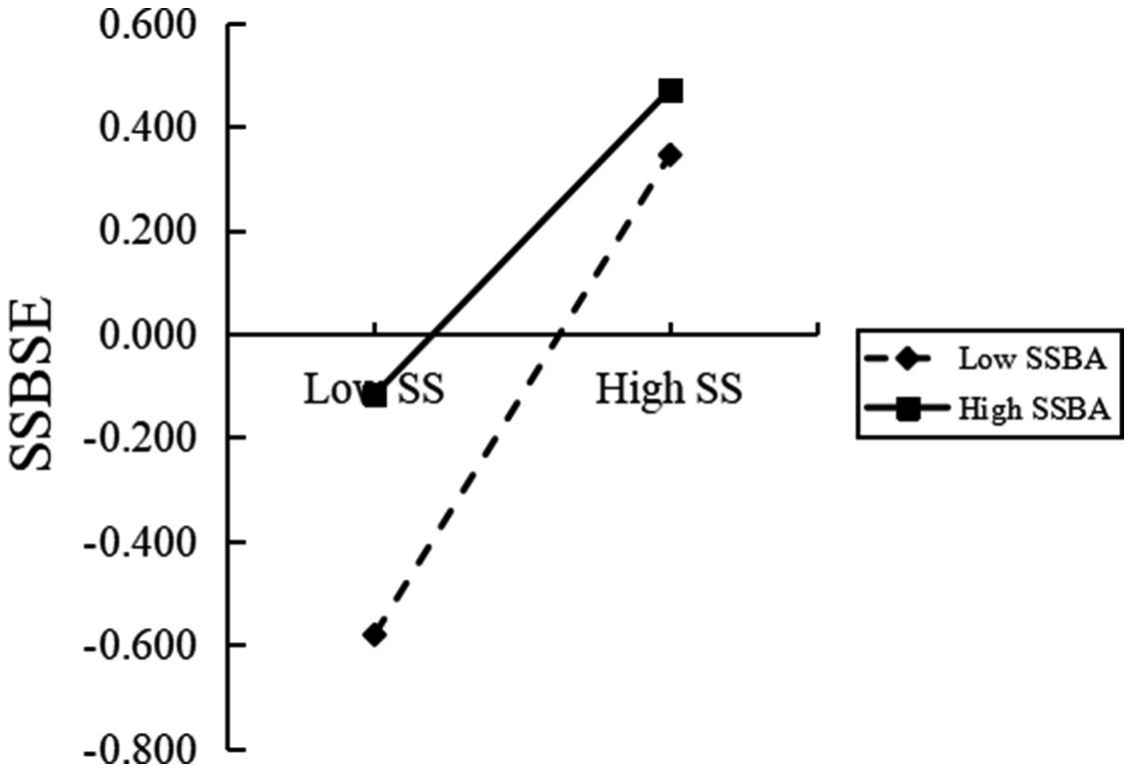
Interaction effect of sport specification behavioral attitude and social support on sports specification self-efficacy.

**Figure 4:**
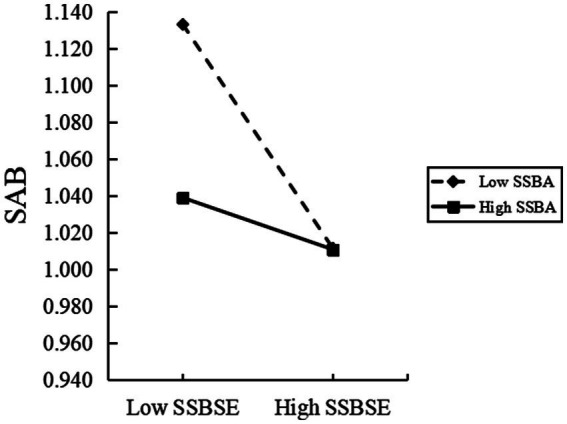
Interaction effect of sports specification self-efficacy and sports specification behavior attitude on sports anomie behavior.

A simple slope analysis is performed successively to further observe the changing trend under regulation, as shown in Figure 3. When the level of sports norm behavior and attitude is low (one standard deviation below the average), social support significantly positively predicts sports norm self-efficacy (*β* simple = 0.2316, *p* < 0.001). When the level of attitude is high (one standard deviation above the average), social support significantly positively predicts physical norm self-efficacy (*β* simple = 0.0630, *p* < 0.05). As shown in Figure 4, when the level of sports standard behavior attitude is low (one standard deviation below the average), sports norm self-efficacy significantly negatively predicts sports anomie behavior (*β* simple = −0.0530, *p* < 0.001). When the attitude of sports norm behavior is high (one standard deviation above the average), the prediction effect of sports norms self-efficacy is not significant (*β* simple = −0.0004, *p* > 0.05).

## 5 Results discussion and countermeasure suggestions

### 5.1 The complete mediation effect of sports specification self-efficacy in college students

According to study results, social support significantly affects college students. Further analysis reveals that when the variable of sports specification self-efficacy is introduced, it completely mediates the relationship between social support and undergraduate PE anomie behavior, confirming Hypothesis H2. This indicates that social support does not directly inhibit sports anomie behavior, but plays an indirect role completely through college students’ self-efficacy. In short, social support indirectly affects college students’ sports anomie behavior by enhancing their confidence in their ability to implement sports behavior norms. Previous studies have shown that self-efficacy plays a completely mediating role in the influence of social support on physical exercise behavior for college students (Wang, 2024). This reveals that the mechanism of action of social support may be far more complex than behavior change alone. It involves deep changes at the psychological level, including psychological processes such as cognitive remodeling, emotional regulation, motivation enhancement, and so on (Huang et al., 2003). Although the perspective that social support may indirectly influence sports anomie behavior in sports by enhancing college students’ self-efficacy in sports norms was proposed, this conclusion is based on the specific sample and measurement tools of this study. Therefore, the mechanism of social support’s role was cautiously interpreted in the discussion. The enhancement effect of social support on the individual’s intrinsic motivation and self-efficacy cannot be ignored. When individuals feel support and encouragement from those around them, they are more likely to believe in their ability to face challenges, which increases their willingness and effort to take positive action. Therefore, social support through the mediated effect at the psychological level influences the individual sports behavior, which is indirect but equally strong and important.

Thoits defines social support as the various help functions provided by important others to individuals, such as family members, friends, colleagues, relatives, and neighbors. These help functions are mainly divided into three categories: social and emotional help, information help, and practical help (He et al., 2020; Troisi and Gabriel, 2011). In the context of physical exercise, the role of social support is particularly significant. Although the sources and types of social support may differently influence college students’ sports behaviors, a more detailed investigation of these aspects was precluded by the limitations of the scale items, suggesting a direction for future research. When individuals encounter setbacks or difficulties, emotional support and encouragement provided by family members, friends, and teachers can stimulate positive emotions, help them overcome challenges, and continue to exercise. This emotional comfort and motivation are important driving forces for individuals to adhere to physical activities.

Information also plays a critical role. Teachers and parents play an important role in educating and guiding health knowledge, exercise information, and behavioral norms. They improve students’ health awareness, willingness to exercise, and self-control by passing on relevant knowledge and setting positive sports behavior examples. This transmission of information and the power of example can enhance individual self-efficacy and encourage participation in physical activity more normatively.

Finally, practical help provides the necessary resources for physical exercise. Social support can help individuals acquire sports equipment and transportation and pay for related activities. It alleviates financial and material barriers that individuals may encounter in physical activity, enabling them to focus more on exercise. The results further confirm the positive effect of social support on the self-efficacy of undergraduate PE norms, thus verifying Hypothesis H2a. As research in sports points out, external support and help can improve the self-efficacy of sports participants and help them better overcome sports difficulties and challenges (White and Bennie, 2015). In conclusion, social support from family members, teachers, and friends positively affects college students’ physical behavior by enhancing their self-efficacy, thus reducing sports anomie behavior. This support gives the individual warmth and strength emotionally and provides necessary support at the information and material level, which jointly promotes standardization of the individual’s sports behavior. It is recommended that universities establish a diversified social support network encompassing family members, friends, and teachers. This can be achieved through organizing regular sports activities, mental health workshops, and team-building activities to enhance interaction and support among students. For instance, implementing a “sports buddy program” could encourage mutual support and monitoring among students, facilitating collective engagement in physical exercise.

Self-efficacy is a precious psychological resource that plays a key role in the mediating relationship between social support and physical behavior (Yang, 2016). Self-efficacy refers to an individual’s confidence in their abilities, which plays a vital role in physical activity. Specifically, individuals with higher self-efficacy are more likely to follow sports norms because they believe in their ability to implement those norms. This belief inspires them to participate in sports activities and enhances their motivation to adhere to sports behavior. Therefore, self-efficacy is a psychological state and an important mechanism to promote individual sports participation and normative compliance. Moreover, data analysis results confirm the negative effect on college students, a finding that supports Hypothesis H2b. This further shows that the higher the self-efficacy of college students’ sports norms, the more positive their attitude and behavior in exercise, and the higher the requirements for their own behavior.

The more positive students’ attitude and behavior in exercise, the higher their behavioral requirements. This positive attitude and high standards reduce the occurrence of sports anomie behavior. Numerous studies support self-efficacy as a mediating variable of behavior change (McAuley et al., 2003), influencing behavior choice, adherence, and level of effort (Yu et al., 2013).

This study’s conclusion reminds us that attention must be paid to improving college students’ self-efficacy in preventing and correcting sports anomie behavior. By enhancing this psychological resource, we can more effectively guide college students to form positive physical education behavior habits, reduce sports anomie behavior, and promote their physical and mental health development. In short, improving college students’ self-efficacy is a key strategy to promote sports behavior norms and prevent sports anomie behavior. It is recommended that universities integrate sports normative education into the physical education curriculum system. By combining theoretical instruction with practical application, students can better understand and adhere to sports norms. For instance, adding a “Sports Norms and Behavior” module to physical education courses, supplemented with case studies and discussions, would help strengthen students’ awareness of normative conduct.

### 5.2 The regulating role of college students’ sports specification behavior attitude

Based on the above conclusions, the self-efficacy of college students’ sports norms completely mediates the influence of social support on college students’ sports anomie behavior. Only the indirect path of sports specification behavior attitude regulation ‘society support→sports specification self-efficacy→sports anomie behavior’ is discussed below. Results prove that the regulatory effect of the sports standard behavior attitude is standardized in the first half and the second half of the path, assuming that hypotheses H3b and H3c are established. Thus, the influence of social support on sports anomie behavior satisfies the moderated mediation model. The results show that social support can indirectly act on sports anomie behavior, and its level of action is regulated by sports norms attitude, among which this fluctuation can be expressed indirectly, through the self-efficacy of sports regulation.

After the model is verified, a simple slope test is conducted to further observe the changing trend of each variable. The simple slope test in Figure 3 shows that in the first half of the fully mediated model, regardless of the high and low sports specification behavior attitude, social support can positively predict college students’ level of sports standard self-efficacy significantly. However, under the attitude of low sports norms, social support is more predictive of college students’ self-efficacy. Previous studies have shown that social support can improve the self-efficacy of college students’ physical exercise, especially the influence of parents and teachers is mediated by self-efficacy (Zhang et al., 2022). Students with a low attitude towards sports standards are more likely to attract the attention of their parents, teachers, friends, and students. Their support and encouragement can significantly improve the self-efficacy of these college students in their sports standard behavior.

The simple slope test in Figure 4 shows that sports-specific behavior attitudes similarly regulate the second half of the fully mediated model, appearing in a different way from the above regulatory process. Specifically, when the level of attitudes toward sports normative behavior is low, an increase in self-efficacy in sports norms demonstrates a significant effect in reducing sports anomie behavior; whereas, when the level of attitudes is high, this effect is not statistically significant. This indicates that the effect of sports-specific self-efficacy on sports anomie behavior is different under different sports-specific attitude levels. According to the conservation of resources theory, injecting new resources is more important for individuals with fewer resources to replenish resources and better resist resource loss. The meaning behind this principle is similar to what we often call “to send charcoal in snowy weather,” that is, the fewer resources individuals have, the more important the injection and increase of resources to relieve tension and pressure. College students with a low attitude towards sports-specific behavior pay more attention to their own behavior because of their low sense of identity for sports-specific behavior, enhancing their ability to self-monitor and self-regulate. This increase in self-awareness helps them more accurately predict whether they will violate sports norms, thus increasing the prediction strength of sports norms’ self-efficacy.

Secondly, individuals with low normative attitudes may have a higher perceived risk of sports specification violations. This risk awareness helps them to improve self-efficacy and thus more effectively prevent and predict sports anomie behavior.

Finally, individuals with low normative attitudes may be more vulnerable to attention and guidance from parents, teachers, and peers. These external supports and feedback can help them improve their self-efficacy and thus more effectively predict and control their behavior. Conversely, individuals who highly identify with sport norms may be overconfident that they are unlikely to violate norms and thus may neglect self-monitoring and self-regulation. Individuals with high normative attitudes may underestimate the risk of norm violation due to overconfidence in norms, and this reduced risk perception may lead to their decreased predictive power of sports anomie behavior.

Finally, individuals with high normative attitudes may need less external feedback because they have already received social recognition and support, which may lead to their decreased ability to predict and control their own behavior. The above research results show that when preventing and reducing sports anomie behavior of college students, we should not only strengthen social support as a relationship of resources, but also pay attention to individual resources, especially the relationship between attitude and self-efficacy. For college students with relatively weak attitudes towards sports-specific behavior, improving their self-efficacy in sports activities is particularly important, as it will prevent and reduce the occurrence of sports anomie behavior more effectively. Universities should establish specialized psychological counseling centers staffed with professional psychological counselors to provide students with psychological support regarding sports behavioral norms and self-efficacy. Regular mental health education courses should be conducted to help students develop positive attitudes toward sports behavior and enhance their sense of self-efficacy. For example, offering a “Sports Psychological Counseling” course could assist students in overcoming psychological barriers in sports activities and strengthening their self-efficacy.

### 5.3 Research contribution and deficiencies

The research contributions of this paper include the selection of research methods. Given the limitations of the traditional Baron-Kenny statistical tests, many studies have pointed out their shortcomings. For example, in analyzing mediation effects, the Baron-Kenny method could not ensure that the independent and the mediation variables on the dependent variable have significant effects, as did the interaction. Moreover, for the treatment of the indirect effects, the Baron-Kenny method requires that the sampling distribution be normally distributed (Hayes, 2009). This assumption is often difficult to meet in practical applications. Given these limitations, this study adopts academic recommendations (Fang and Zhang, 2012) and uses the Process plug-in components combined with the BC-Bootstrap interval estimation method to analyze data. This method can provide more accurate and reliable results, overcoming the shortcomings of traditional methods. Secondly, though current academic research on social support and physical exercise behavior promotion is relatively abundant, discussion of the influence mechanism of social support on college students’ sports anomie behavior is relatively insufficient, especially in quantitative research. This paper fills this gap through an empirical analysis method, enriching the theoretical basis of the relationship between social support and college students and its influence mechanism. It also provides practical guidance to schools in preventing and reducing college student issues.

Finally, this study thoroughly investigated the mechanism through which social support influences college students’ sports anomie behavior. Through mediation effect analysis, it was found that social support, to some extent, affects such behavior and effectively reduces its occurrence. Furthermore, the analysis of the regulation effect confirms that the attitude of sports norm behavior plays a positive role in regulating social support and the self-efficacy of sports norm behavior, as well as between the self-efficacy of sports regulation and sports anomie behavior. When college students hold a more positive attitude towards sports normative behavior, social support more significantly affects the self-efficacy of sports normative behavior, reducing sports anomie behavior. This finding highlights the importance of social support and positive attitude in promoting the standardization of sports behavior among college students.

Shortcomings and prospects of this study: This study comprehensively investigates the mechanism through which social support, self-efficacy in sports norms, attitudes toward sports normative behavior, and sports anomie behavior interact, providing valuable insights for understanding this field. However, several limitations should be noted:

(1) Limitations of measurement tools: Although the revised social support scale used in this study considered the sources of support, its one-dimension restricted an in-depth exploration of social support. Future research could adopt or develop localized, multidimensional social support scales to more comprehensively assess various aspects of social support.
(2) Small effect sizes: The effect sizes observed in this study were relatively small, which may be related to the research design, sensitivity of measurement tools, and omission of potential influencing factors. Future studies could optimize research designs, employ more sensitive measurement instruments, and consider other potential factors (e.g., motivation, physical condition, sports participation behavior) to improve the estimation of effect sizes.
(3) Limitations of research methodology: This study employed a cross-sectional design, which limited the ability to reveal dynamic relationships among variables. Future research could adopt longitudinal or experimental approaches to further empirically analyze the relationships between social support, self-efficacy in sports norms, attitudes toward sports normative behavior, and sports anomie behavior. Such methods would enable a deeper understanding of the dynamic interactions among these variables and provide stronger evidence for related research.

## Data Availability

The raw data supporting the conclusions of this article will be made available by the authors, without undue reservation.
