# Supplementary figures and images for: The effect of social support on college students’ sports anomie behavior: the mediating role of self-efficacy and attitude’s regulating role in sports normative behavior
Source: Front Psychol. 2025 Sep 25;16:1662985. doi: 10.3389/fpsyg.2025.1662985 (PMC12511870; doi:10.3389/fpsyg.2025.1662985)

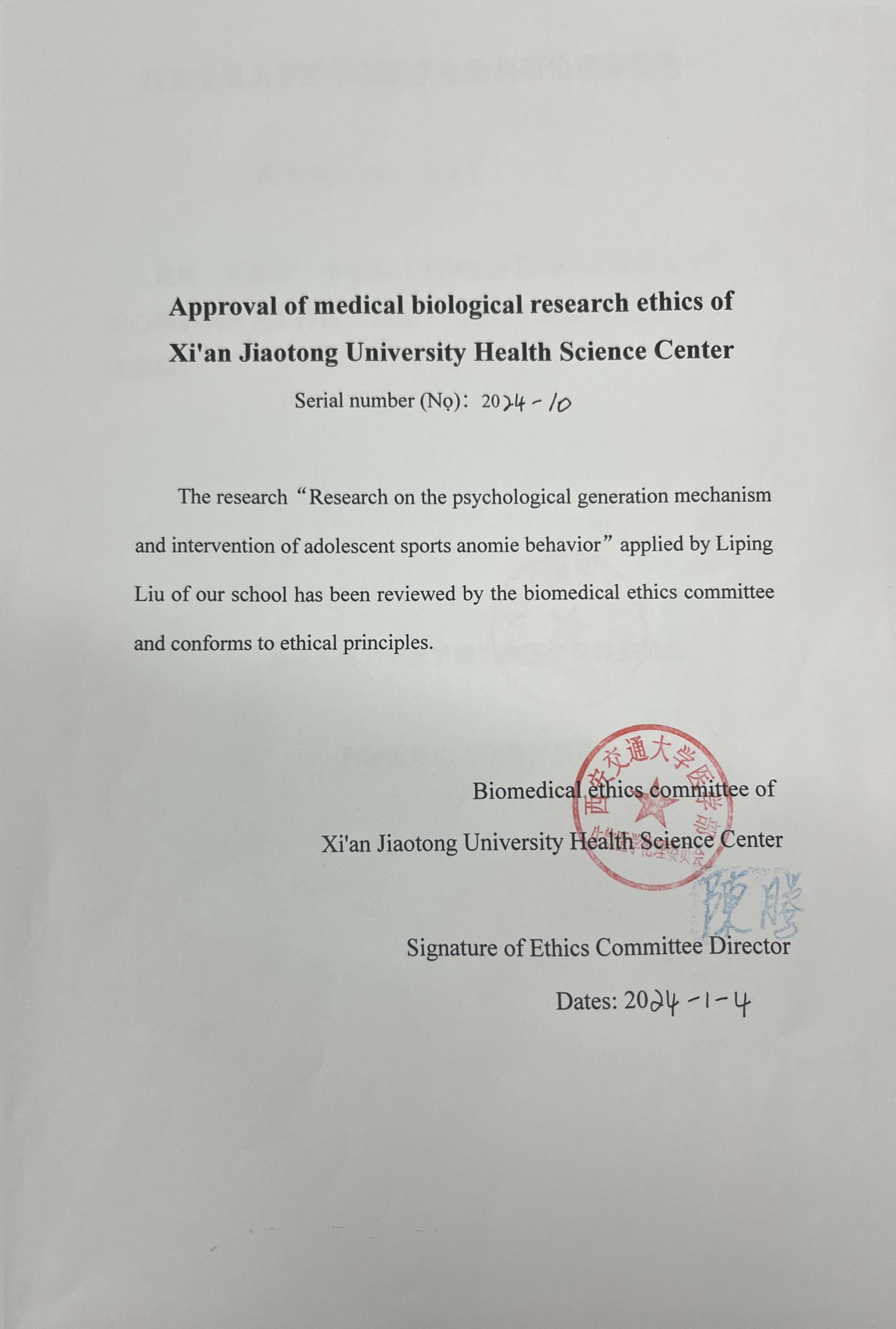

Supplement: Supplementary file 1 [file Data_Sheet_1.zip › Supplementary files /Ethical Document (English Version).jpg]

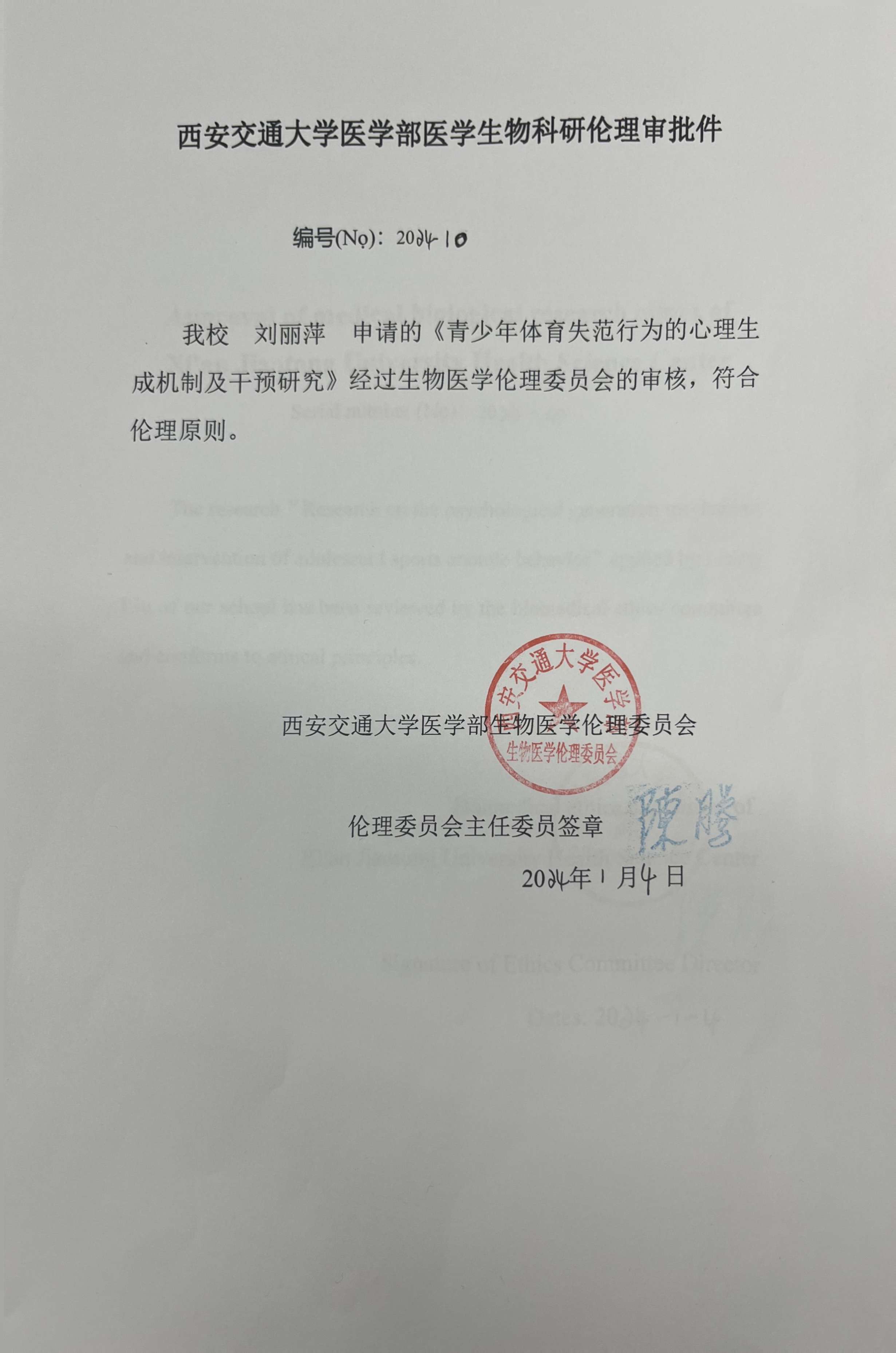

Supplement: Supplementary file 1 [file Data_Sheet_1.zip › Supplementary files /Ethical Document (Original).jpg]
